# Dietary Fat Intake Modulates Effects of a Frequent *ACE* Gene Variant on Glucose Tolerance with association to Type 2 Diabetes

**DOI:** 10.1038/s41598-017-08300-7

**Published:** 2017-08-23

**Authors:** Rita Schüler, Martin A. Osterhoff, Turid Frahnow, Matthias Möhlig, Joachim Spranger, Darko Stefanovski, Richard N. Bergman, Li Xu, Anne-Cathrin Seltmann, Stefan Kabisch, Silke Hornemann, Michael Kruse, Andreas F. H. Pfeiffer

**Affiliations:** 1 Department of Clinical Nutrition, German Institute of Human Nutrition Potsdam-Rehbrücke (DIfE), Nuthetal, Germany; 2 German Center for Diabetes Research (DZD), München-Neuherberg, Germany; 3 Department of Endocrinology, Diabetes and Nutrition, Charité-Universitätsmedizin Berlin, Campus Benjamin Franklin, Berlin, Germany; 4 Charité-Center for Cardiovascular Research (CCR), Charité-Universitätsmedizin Berlin, Berlin, Germany; 5 German Center for Cardiovascular Research (DZHK), Partner Site Berlin, Berlin, Germany; 6 New Bolton Center, School of Veterinary Medicine, University of Pennsylvania, Philadelphia, Pennsylvania USA; 7 Diabetes and Obesity Research Institute, Cedars-Sinai Medical Center, Los Angeles, California, USA

## Abstract

The frequent *ACE* insertion/deletion polymorphism (I/D) is, albeit inconsistently, associated with impaired glucose tolerance and insulin resistance. We recently observed an enhanced upregulation of ACE by elevated fat intake in *GG*-carriers of the I/D-surrogate rs4343 variant and therefore investigated its potential nutrigenetic role in glucose metabolism. In this nutritional intervention study 46 healthy and non-obese twin pairs consumed recommended low fat diets for 6 weeks before they received a 6-week high fat (HF) diet under isocaloric conditions. Intravenous glucose tolerance tests were performed before and after 1 and 6 weeks of HF diet. While glucose tolerance did not differ between genotypes at baseline it significantly declined in *GG*-carriers after 6 weeks HF diet (*p* = 0.001) with higher 2 h glucose and insulin concentrations compared to *AA/AG*-carriers (*p* = 0.003 and *p* = 0.042). Furthermore, the gene-diet interaction was confirmed in the cross-sectional Metabolic Syndrome Berlin Potsdam study (*p* = 0.012), with the GG-genotypes being significantly associated with prevalent type 2 diabetes for participants with high dietary fat intake ≥37% (*GG* vs. *AA/AG*, OR 2.36 [1.02–5.49], *p* = 0.045). In conclusion, the association between the rs4343 variant and glucose tolerance is modulated by dietary fat intake. The *ACE* rs4343 variant is a novel nutrient-sensitive type 2 diabetes risk marker potentially applicable for nutrigenetic dietary counseling.

## Introduction

A large body of evidence supports the role of the renin-angiotensin system (RAS) in modulation of glucose metabolism and its involvement in insulin resistance^1^. Inhibition of angiotensin-converting enzyme (ACE), and thereby antagonizing the RAS, has been shown to improve glucose homeostasis and, albeit inconsistently, to reduce incidence of type 2 diabetes mellitus in large-scale clinical trials^1–7^.

Furthermore, the D allele of the frequent *ACE* insertion/deletion (I/D) polymorphism, which is characterized by the presence (I) or absence (D) of a 287-bp *Alu* repeat sequence in the 16^th^ intron of the *ACE* gene, was associated with decreased insulin sensitivity and impaired glucose tolerance in a healthy cohort^8^.

Circulating ACE levels were shown to increase in obesity and to decrease during weight loss^9–12^. Recently, we identified *ACE* as nutrition-responsive gene in the NUtriGenomic Analysis in Twins (NUGAT) study, with increased ACE concentrations in response to an isocaloric high fat diet in healthy and non-obese subjects independent of weight gain^13^. Therefore, we intended to investigate potential effects of a high fat diet on glucose tolerance as well as insulin sensitivity in our NUGAT study dependent on the frequent *ACE* rs4343 variant, a surrogate marker for the I/D polymorphism, where the A allele corresponds to the I allele and the G allele corresponds to the D allele^14^. Additionally, the cross-sectional Metabolic Syndrome Berlin Potsdam (MeSyBePo) study, which includes nutritional assessments, was analyzed to evaluate the influence of dietary fat intake on the association between *ACE* genotype and type 2 diabetes prevalence in order to complement and validate the analysis in the NUGAT study.

## Results

### NUGAT study

The main clinical characteristics at screening of the 92 subjects studied are presented in Table 1. Rs4343 genotype frequencies were distributed according to Hardy-Weinberg equilibrium (Table 1). At screening, no significant differences in markers of glucose metabolism and insulin sensitivity were observed between rs4343 genotypes (*p*_fasting insulin_ = 0.275, *p*_fasting glucose_ = 0.659, *p*_HOMA-IR_ = 0.204, *p*_HbA1c_ = 0.128, Table 1).

**Table 1:** Characteristics of the participants overall and stratified for *ACE* rs4343 at baseline in the NUtriGenomic Analysis in Twins study.

	Total	*AA* Genotype	*AG* Genotype	*GG* Genotype	*p*-Value
n	92	31	44	17	0.842
Male/Female	34/58	10/21	14/30	10/7	0.120
Age (years)	31 ± 14	30 ± 14	31 ± 11	34 ± 20	0.764
BMI (kg/m²)	22.8 ± 2.7	22.8 ± 2.2	22.9 ± 2.7	22.9 ± 3.6	0.990
Fasting insulin (mU/l)	5.21 ± 3.68	4.93 ± 3.04	4.91 ± 3.36	6.51 ± 5.23	0.275
Fasting glucose (mmol/l)	4.31 ± 0.43	4.32 ± 0.43	4.26 ± 0.41	4.37 ± 0.51	0.659
HOMA-IR	1.01 ± 0.76	0.95 ± 0.59	0.94 ± 0.65	1.31 ± 1.16	0.204
HbA_1c_ (%)	5.0 ± 0.4	5.1 ± 0.5	4.9 ± 0.3	5.0 ± 0.2	0.128

Values are shown as mean ± SD.

**Figure 1:**
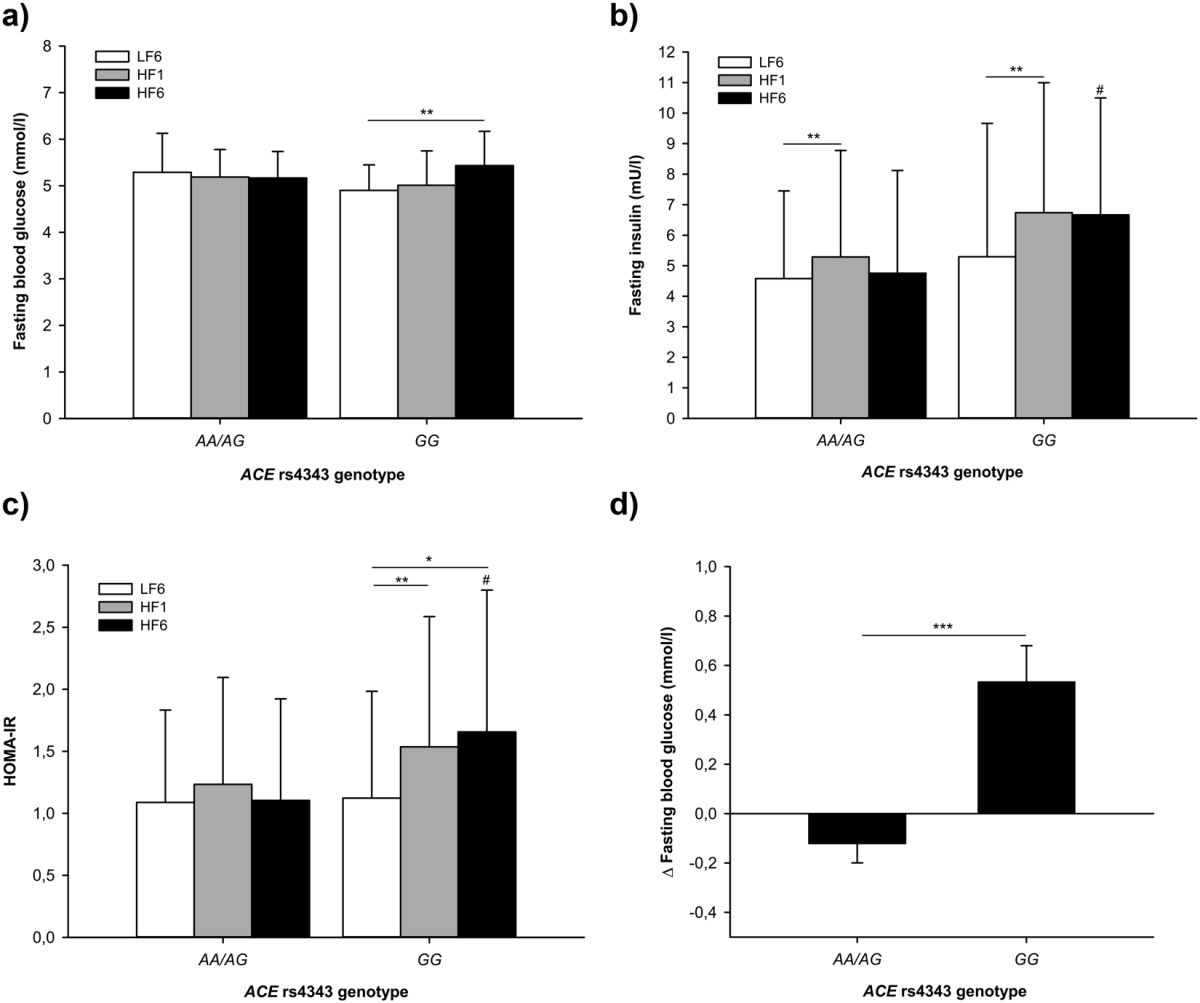
Measured and calculated parameters of glucose metabolism at LF6, HF1 and HF6 stratified by *ACE* rs4343 genotype (recessive model): (**a**) Fasting blood glucose, (**b**) ∆ Fasting blood glucose (HF6-LF6; mean ± SEM), (**c**) Fasting insulin and (d) HOMA-IR. Data are shown as mean ± SD; *p < 0.05, **p < 0.01, ***p < 0.001, ^#^ *p* < 0.05 vs. *AA/AG* at HF6. LF6: CID after 6 weeks of LF diet; HF1/HF6: CID after 1/6 weeks of HF diet.

During HF diet intervention (Table 2), fasting glucose values and glucose tolerance as assessed by ivGTT did not change, whereas fasting insulin values (repeated measures ANOVA; *p* = 0.006) and HOMA-IR increased (repeated measures ANOVA; *p* = 0.012) with significant differences in response to 1 week of HF diet (Bonferroni *posthoc* analysis; LF6 (clinical investigation day (CID) after 6 weeks of carbohydrate-rich low-fat diet) vs. HF1 (CID after 1 week of high fat diet): *p*_fasting insulin_ = 2.4 × 10^−4^, *p*_HOMA-IR_ = 0.002). However, we observed a significant rs4343 genotype × HF intervention interaction on fasting glucose levels (repeated measures ANOVA, interaction term rs4343 (recessive model) × HF intervention: *p* = 0.001, *p* = 0.004 adjusted for sex, age and BMI). Stratified by rs4343 genotype (recessive model), we observed a significant increase in fasting blood glucose concentrations in *GG*-carriers by 0.5 ± 0.1 mmol/l (mean ± SEM), whereas no change was observed for *AA/AG*-carriers (repeated measures ANOVA; *p*_*AA/AG*_ = 0.191 *vs*. *p*_*GG*_ = 0.009; Fig. 1a). Changes in fasting blood glucose in response to the HF diet also significantly differed between genotypes (Fig. 1b, *p* = 4.5 × 10^−4^). As shown in Fig. 1c, fasting insulin concentrations significantly increased irrespective of genotypes (repeated measures ANOVA, *p*_*AA/AG*_ = 0.038 and *p*_*GG*_ = 0.039; interaction term rs4343 (recessive model × HF intervention: *p* = 0.175), with significant differences in response to 1 week of HF diet (Bonferroni *posthoc* analysis *p*_*AA/AG*_ = 0.008 and *p*_*GG*_ = 0.005). However, after 6 weeks of HF diet fasting insulin concentrations were significantly higher in *GG*-carriers compared to *AA/AG*-carriers (*p* = 0.042). A significant interaction between rs4343 genotype and the HF intervention was also observed on HOMA-IR (repeated measures ANOVA, interaction term rs4343 (recessive model) × HF intervention: *p* = 0.008, *p* = 0.028 adjusted for sex, age and BMI). As shown in Fig. 1d, HOMA-IR values increased in *GG*-carriers (repeated measures ANOVA *p*_*GG*_ = 0.002) with significantly higher concentrations at HF6 (CID after 6 weeks of high fat diet) compared to *AA/AG*-carriers (*AA/AG vs*. *GG p* = 0.022), whereas HOMA-IR measures did not change for *AA/AG* genotypes during HF diet (repeated measures ANOVA *p*_*AA/AG*_ = 0.075).

**Table 2:** Characteristics of the participants (n = 92) after the standardization (LF6) and after 1 and 6 weeks of high-fat diet (HF1, HF6).

	LF6	HF1	HF6	*p*-Value
Weight (kg)	66.6 ± 11.7	66.5 ± 11.6	67.0 ± 11.8*	9 × 10^−6^
BMI (kg/m²)	22.5 ± 2.7	22.5 ± 2.6	22.6 ± 2.7*	1.1 × 10^−5^
Fasting glucose (mmol/l)	5.22 ± 0.81	5.15 ± 0.62	5.22 ± 0.61	0.550
Fasting insulin (mU/l)	4.71 ± 3.19	5.55 ± 3.66*	5.11 ± 3.52	0.006
HOMA-IR	1.09 ± 0.76	1.29 ± 0.90^#^	1.21 ± 0.91	0.012
2-h glucose (mmol/l)	4.46 ± 0.69	4.38 ± 0.64	4.43 ± 0.64	0.707
iAUC_glucose_ (mmol l^−1^ min^−1^)	213 ± 71	215 ± 51	219 ± 72	0.548
iAUC_insulin_ (mU l^−1^ min^−1^)	1748 ± 970	1890 ± 1346	1780 ± 1029	0.235

Values are shown as mean ± SD. Repeated measures ANOVA with Bonferroni *posthoc* test: *p < 0.001 and ^#^ *p* < 0.01 com*p*ared to LF6. iAUC, incremental area under the curve.

**Figure 2:**
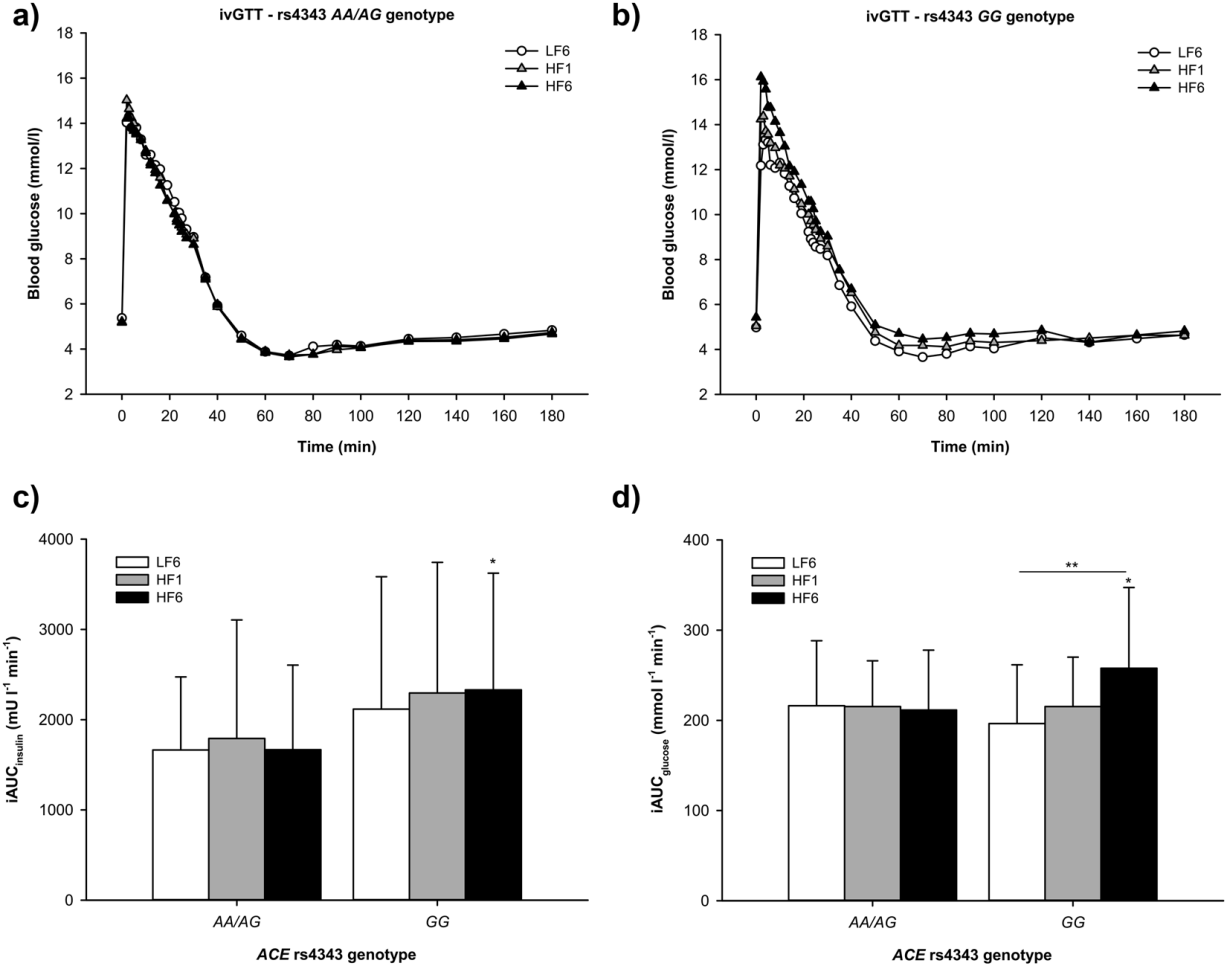
Blood glucose concentrations during ivGTT in (**a**) *AA/AG*-carriers and (**b**) *GG*-carriers. Increments of (**c**) blood glucose and (**d**) insulin at LF6, HF1 and HF6 (mean ± SD; *p < 0.05 vs. *AA/AG*-carriers at HF6).

A significant genotype × intervention interaction was also revealed for the incremental area under the curve (iAUC) for glucose during ivGTT (repeated measures ANOVA, interaction term rs4343 (recessive model) × HF intervention: *p* = 0.014, *p* = 0.025 adjusted for sex, age and BMI). In response to a 6-week isocaloric high fat diet, incremental area under the curve (iAUC) for glucose during ivGTT significantly increased in *GG*-carriers (Fig.  2b and c; repeated measures ANOVA, *p*_iAUCglucose_ = 0.001), whereas no change was observed for *AA/AG*-carriers (repeated measures ANOVA, *p*_iAUCglucose_ = 0.941; ∆iAUC_glucose_ LF6-HF6, *AA/AG* vs. *GG*: *p* = 0.009; *AA/AG* vs. *GG* iAUC_glucose_: *p*_LF6_ = 0.330, *p*_HF1_ = 0.999, *p*_HF6_ = 0.028). iAUC for insulin was also significantly higher in *GG*-carriers after 6 weeks of HF diet compared to *AA/AG*-carriers (Fig. 2d, *p*_iAUCinsulin_ = 0.027). Furthermore, *GG*-carriers responded with higher 2 h glucose levels during ivGTT compared to AA/AG-carriers (*GG*: 4.8 ± 0.7 mmol/l; *AA/AG*: 4.3 ± 0.6 mmol/l; *p* = 0.003).

Indices for insulin sensitivity (Si), glucose effectiveness (Sg) and diposition index (DI) did not differ significantly between the genotypes (*AA/AG* vs. *GG*; Si_HF6_ 11.6 ± 7.7 vs. 8.8 ± 5.3 (mU/l)^−1^ × min^−1^, *p*_HF6_ = 0.204; Sg_HF6_ 2.09 ± 1.03 vs. 1.97 ± 0.95 × 100 min^−1^, *p*_HF6_ = 0.652; DI_HF6_ 2759 ± 1708 vs. 3311 ± 2377, *p*_HF6_ = 0.317) and did not change significantly in response to HF diet. The acute insulin response to glucose (AIRg) significantly differed between genotypes at HF1 and HF6 (*AA/AG* vs. *GG*; AIRg_LF6_ 236 ± 118 vs. 298 ± 159 mU × l^−1^ × min, *p*_LF6_ = 0.081; AIRg_HF1_ 244 ± 111 vs. 340 ± 121 mU × l^−1^ × min, *p*_HF1_ = 0.003; AIRg_HF6_ 257 ± 140 vs. 363 ± 155 mU × l^−1^ × min, *p*_HF6_ = 0.010).

## Metabolic Syndrome Berlin Potsdam (MeSyBePo) study

To examine dietary fat intake dependent effects, we stratified subjects into high fat diet consumers with energy from total fat ≥37% (cutoff value represents the average fat intake in western countries)^15^ and normal/low fat diet consumers (<37% energy from fat). Genotype frequencies did not deviate from Hardy-Weinberg equilibrium (irrespective of fat intake *p* = 0.085, <37% *p* = 0.074, ≥37% *p* = 0.584).

Irrespective of dietary fat intake no association between the rs4343 variant and type 2 diabetes was found (recessive model: OR 1.11 [0.61–2.02], *p* = 0.736 adjusted for age, sex and BMI). After stratification by fat intake, a significant association of rs4343 polymorphism with type 2 diabetes was observed in subjects with a reported dietary fat intake ≥37% with a 2.7-fold higher risk for type 2 diabetes for *GG*-carriers as compared to *AA/AG*-carriers (*p* = 0.035 after adjustment for age, sex and BMI), whereas for normal fat intake no association was found (Table 3). Furthermore, *GG*-carriers with high dietary fat intake had 4.6-fold increased risk for type 2 diabetes as compared to *GG*-carriers with normal dietary fat intake (recessive model: OR 4.61 [1.58–13.46], *p* = 0.005 adjusted for age, sex and BMI). Also a multiplicative interaction term between genotype and dietary fat intake was significant (recessive model: OR 1.45 [1.09–1.94], *p* = 0.012 adjusted for age, sex and BMI).

**Table 3:** Association between *ACE* rs4343 genotype and type 2 diabetes in the cross-sectional MeSyBePo cohort

	Fat intake < 37%		Fat intake ≥ 37%	
	*AA/AG*	*GG*	*AA/AG*	*GG*
n_control_/n_case_	146/27	64/8	80/20	23/13
OR (95% CI)	1 (ref.)	0.60 (0.25–1.47)	1 (ref.)	2.71 (1.07–6.84)
*p* value	—	0.267	—	0.035

Binary logistic regression model adjusted for age, sex and BMI. n_control_/n_case_, participants without/with type 2 diabetes.

## Discussion

The results of the NUGAT study showed that dietary fat intake decisively modulated the association of the *ACE* rs4343 variant with impaired glucose metabolism and insulin resistance in healthy and non-obese subjects. Furthermore, we observed in the cross-sectional MeSyBePo cohort that the association between the rs4343 variant and prevalent type 2 diabetes was also influenced by dietary fat intake, with an increased risk for type 2 diabetes in *GG*-carriers of the rs4343 polymorphism provided that dietary fat intake was ≥37%.

The rs4343 variant is in nearly perfect linkage disequilibrium with the *ACE* I/D polymorphism^16^, whereby the A-allele corresponds to the insertion (I) variant and the G-allele to the deletion (D) variant, which is characterized by higher circulating enzyme levels^17^. With regard to associations between the *ACE* I/D polymorphism and glucose metabolism, results have been contradictory. While in a study of Bonnet *et al*. healthy subjects homozygous for the D-allele were shown to have decreased insulin sensitivity as measured via clamp and increased 2 h plasma glucose concentrations during OGTT, they and others reported no association between the *ACE* I/D variant and fasting concentrations of insulin and glucose in non-diabetic subjects^8, 18, 19^. Studies investigating the association of the I/D variant with risk for type 2 diabetes have also yielded conflicting results^20–22^. However, two meta-analyses demonstrated an increased type 2 diabetes risk for the D-allele^23, 24^. Also treatment with ACE inhibitors is associated with improvements in glucose metabolism and, albeit inconsistently, reduced incidence of mellitus in large-scale clinical trials^1–7^.

With regard to our observations in healthy subjects in the NUGAT study, we found no association per se between glucose tolerance assessed as increments of glucose and insulin during ivGTT as well as fasting glucose and insulin concentrations confirming previous studies with regard to the I/D variant. Nevertheless, after 6 weeks of challenge with a HF diet significantly impaired values for glucose and insulin concentration could be observed, demonstrating that a nutritional challenge influences the effect of the genotype on those parameters. Also in our second, independent MeSyBePo cohort, type 2 diabetes was more prevalent among *GG*-carriers given that dietary fat intake was high. Our results show that consideration of dietary fat intake may be required to elucidate effects of *ACE* rs4343 on glucose metabolism and may explain inconsistent results of previous studies.

The NUGAT study is limited by the relatively small number of participants with respect to genotype-stratified data analysis. Nonetheless, confounding was reduced by including only metabolically healthy, normotensive, non-obese and rather young participants into the study. Moreover, all participants were Caucasians; therefore applicability to other non-Caucasian ethnicities needs to be elucidated. In general, the molecular mechanism by which altered ACE concentrations, *ACE* genotype and ACE inhibitors affect glucose metabolism require clarification, as they are poorly understood. A broadly based understanding will create the basis to elucidate the interplay between ACE and dietary fat and its impact on glucose metabolism. Another aspect which deserves further investigation is the quality of fat. The high fat dietary pattern in the NUGAT study was characterized by the emphasis on saturated fats (18% of 45% energy from total fat) from meat and whole-milk products. Dairy fat intake was inversely associated with markers of glucose metabolism in a Swedish study^25^. Therefore, it would be important to evaluate the effects of fat quality on the gene-diet interaction.

Our data showed that markers of glucose metabolism and type 2 diabetes risk were significantly influenced by interactions between the *ACE* rs4343 variant and dietary fat intake and suggested that homozygous carriers of the G-allele responded unfavorably to high fat diets with increased risk for altered glucose metabolism and type 2 diabetes.

## Methods

### NUGAT study design

The NUGAT study was approved by the independent ethics review committee of the Charité-Universitätsmedizin Berlin and conducted in accordance with the principles of the Helsinki Declaration of 1975, as revised in 2000. All participants provided written informed consent prior to the study.

Details of recruitment and phenotyping of study participants as well as dietary interventions were published recently^13^. 46 healthy and non-obese twin pairs (34 mono- and 12 dizygotic pairs; 58 female and 34 male subjects) with a mean age of 31 ± 14 years and a mean BMI of 22.8 ± 2.7 kg/m² were included in the study. At screening a standardized 3 h, 75 g OGTT (oral glucose tolerance test) with insulin measurements was performed.

The dietary intervention was carried out in a sequential design and under isocaloric conditions. Individual energy requirements were calculated based on participants resting energy expenditure (REE) determined by indirect calorimetry and physical activity level assessed by questionnaire. Participants were standardized for their nutritional behavior prior to the study via a 6-week carbohydrate-rich low-fat diet (LF, 55% carbohydrate, 30% fat, 15% protein) before they switched to a 6-week HF diet (40% carbohydrate, 45% fat, 15% protein) with emphasis on foods high in saturated fat. Participants were given intensive, regular and detailed dietary guidance by a nutritionist over the entire period of intervention to ensure compliance. Furthermore, all participants had to complete 5 dietary records during the 12 weeks of the dietary intervention period. Dietary protocols had been analyzed via Software PRODI 4.5 LE 2001 Expert (Firma Nutri-Science, Hausach, Germany) to quantify energy and macronutrient composition to ensure the adherence to the dietary phases.

Clinical investigation days (CIDs) were performed after 6 weeks of LF diet (LF6) and after 1 and 6 weeks of HF diet (HF1 and HF6). At each CID fasting blood glucose and insulin concentrations were measured and intravenous glucose tolerance tests were performed.

### Intravenous glucose tolerance test (ivGTT)

After 12 hours of fasting a bolus of glucose (11.4x body surface area/0.5 ml) was infused as a 50% (w/v) solution (Braun, Melsungen, Germany) intravenously within 1 min. Afterwards physiologic saline solution was injected in order to prevent phlebitis. After 20 min, an insulin bolus of 0.03 U per kg body weight was injected (InsumanRapid 40 IU/ml, Sanofi-Aventis, Frankfurt, Germany). Blood for measurement of insulin and glucose concentration was drawn from a peripheral venous access catheter placed into a forearm vein at 0, 2, 3, 4, 5, 6, 8, 10, 12, 14, 16, 19, 22, 23, 24, 25, 27, 30, 35, 40, 50, 60, 70, 80, 90, 100, 120, 140, 160 and 180 min.

We applied the linear trapezoid rule to calculate incremental areas under the curve (iAUC) for plasma glucose and insulin. Indices for insulin sensitivity (Si), glucose effectiveness (Sg), acute insulin response to glucose (AIRg) and disposition index (DI) were calculated using the minimal model (MINMOD Millenium)^26^.

### Blood Parameters

Blood glucose was measured by the glucose oxidase method (Super GL, Dr. Müller Gerätebau, Freital, Germany). Insulin was determined in serum by ELISA (Mercodia, Uppsala, Sweden). Homeostasis model assessment of insulin resistance (HOMA-IR; [(fasting insulin (mU/l))×(fasting glucose (mmol/l))/22.5]) was calculated to determine basal/hepatic insulin sensitivity.

### Rs4343 polymorphism genotyping

In the NUGAT study, genomic DNA was extracted from buffy coat samples (NucleoSpin, Macherey-Nagel, Düren, Germany) and genotyped using HumanOmniExpressExome BeadChips (Illumina, Inc., San Diego, CA, USA) at the Interdisciplinary Center for Clinical Research (IZKF, Leipzig, Germany). For the Metabolic Syndrome Berlin Potsdam study, genotyping was performed using a predesigned TaqMan SNP Genotyping Assay (ViiA7 System; Applied Biosystems, Foster City, CA).

### Metabolic Syndrome Berlin Potsdam Study

The cross-sectional Metabolic Syndrome Berlin Potsdam (MeSyBePo) study was approved by the ethics commissions of Berlin and Brandenburg, Germany. All participants provided written informed consent.

The study included 2364 participants, who were randomly recruited from Berlin, Potsdam and surroundings, Germany. All participants underwent physical examination and fasting blood test, as well as a standardized 3-h 75-g OGTT (unless no evident diagnosis of diabetes was existent) with insulin measurements in nondiabetic subjects. Diabetes was diagnosed considering the ADA criteria from 2002^27^. Dietary information was assessed by a 4-day estimated food record which comprised 18 categories with 151 food items. Means of daily energy and nutrient intakes were calculated on the basis of the German Nutrient Database BLS version 2.3. Misreporting of dietary intake was evaluated as recently published^13^ and only subjects who reported normal energy intakes were included in the analyses. A complete set of data including dietary information (given normal dietary reporting) was available from 381 participants (305 females and 76 males, mean age 53.4 years, mean BMI 28.7 kg/m^2^).

### Statistical Analysis

Variables were assessed for normal distribution using the Kolmogorov-Smirnov test and were natural logarithm (ln)-transformed in case of skewed distribution. To compare mean values for continuous data one-way or repeated measures ANOVA followed by Bonferroni *posthoc* test was used. Analyses were adjusted for potential confounding variables such as sex, age and BMI. Significant results for non-normally distributed data were verified using the Kruskal-Wallis test as non-parametric equivalent of the ANOVA.

Genotype frequencies were analyzed for deviation from Hardy-Weinberg equilibrium by chi-square test using R 3.1.2 plus HardyWeinberg package 1.5.5. Chi-Square test was used to study associations between rs4343 genotype and type 2 diabetes frequencies. To estimate odds ratios in the MeSyBePo cohort unconditional logistic regression analysis was performed, also with adjustment for age, sex and BMI. Statistical significance was designated at *p* < 0.05. Values are expressed as mean ± SD, unless otherwise stated. SPSS 20.0 (SPSS Inc., Chicago, IL, USA) was used for statistical analyses.
